# Alcohol consumption and PSA-detected prostate cancer risk—A case-control nested in the ProtecT study

**DOI:** 10.1002/ijc.27877

**Published:** 2012-10-25

**Authors:** Luisa Zuccolo, Sarah J Lewis, Jenny L Donovan, Freddie C Hamdy, David E Neal, George Davey Smith

**Affiliations:** 1 MRC Centre for Causal Analyses in Translational Epidemiology, University of BristolBristol, United Kingdom; 2 School of Social and Community Medicine, University of BristolBristol, United Kingdom; 3 Nuffield Department of Surgical Sciences, Uro-oncology group, University of OxfordOxford, United Kingdom; 4 Department of Oncology, University of CambridgeCambridge, United Kingdom

**Keywords:** alcohol, prostate cancer, prostate specific antigen, ProtecT, nested case–control

## Abstract

Alcohol is an established carcinogen but not an established risk factor for prostate cancer, despite some recent prospective studies suggesting increased risk among heavy drinkers. The aim of this study was to investigate the role of alcohol on prostate-specific antigen (PSA) levels and prostate cancer risk. Two thousand four hundred PSA detected prostate cancer cases and 12,700 controls matched on age and general practice were identified through a case-control study nested in the PSA-testing phase of a large UK-based randomized controlled trial for prostate cancer treatment (ProtecT). Linear and multinomial logistic regression models were used to estimate ratios of geometric means (RGMs) of PSA and relative risk ratios (RRRs) of prostate cancer by stage and grade, with 95% confidence intervals (CIs), associated with weekly alcohol intake and drinking patterns. We found evidence of lower PSA (RGM 0.98, 95% CI: 0.98–0.99) and decreased risk of low Gleason-grade (RRR 0.96; 95%CI 0.93–0.99) but increased risk of high-grade prostate cancer (RRR 1.04; 95%CI 0.99–1.08; *p*_difference_=0.004) per 10 units/week increase in alcohol consumption, not explained by current BMI, blood pressure, comorbidities, or reverse causation. This is the first large population-based study to find evidence of lower PSA levels for increasing alcohol consumption, with potential public health implications for the detection of prostate cancer. Our results also support a modestly higher risk of high-grade disease for heavy drinkers, but require independent replication to establish the nature of the association of alcohol with low-grade disease, preferably in cohorts with a heterogeneous case-mix.

**What's new?:**

Alcohol is not an established risk factor for prostate cancer; however, the current work suggests that heavy drinking could cause a small increase in risk of the more aggressive forms. If the results are confirmed to be causal, prostate cancer risk will be added to the many long-term health risks of heavy drinking, and public health strategies will then also reduce high-risk, poorer prognosis prostate cancer. The authors also found that heavy drinkers have lower PSA levels, suggesting that heavy alcohol consumption could be used as a marker to identify men in whom some cancers might be missed.

Alcohol is a known human carcinogen,^1^ but there is limited evidence on whether it affects prostate cancer.^2^ Alcohol use and abuse are common in developed countries like the UK and USA and are rapidly increasing in many developing countries, especially amongst the urban poor.^3^ More research into a possible causal link between alcohol and prostate cancer is justified by the potential to discover a target for disease prevention on the one hand, and on the other by the need to better characterize the long-term effects of alcohol consumption over the life-course.

The evidence from epidemiological studies on the role alcohol plays in prostate cancer is inconclusive, despite the many publications to date, including 14 prospective studies, which have been pooled into one of two meta-analyses,^4^,^5^ and at least six new studies published after the searches for those two reviews were last updated.^6^–^11^ The two meta-analyses based on systematic reviews yielded remarkably similar pooled estimates, with random-effects relative risks of 1.02 (95% confidence interval (CI): 0.85–1.23)^4^ per additional drink/day, and of 1.03 (95% CI: 1.00–1.07)^5^ per additional unit/day. Three of the six most recent prospective studies published after these review found some evidence of an increased risk of high-grade disease ranging from a 20% increase to a doubling of the risk in the heaviest drinkers (consuming 1+ to 5+ drinks/day) as compared to light or nondrinkers,^7^–^9^ whereas two studies, one on advanced and fatal disease,^11^ did not find increased risks,^6^,^11^ and another did not report results for heavy drinkers.^10^ Evidence from the Prostate Cancer Prevention Trial of an interaction between heavy drinking and the antiandrogen finasteride further suggests the possibility of a real and important effect of alcohol on prostate carcinogenesis.^7^

Although the available evidence does not offer any clear indication for a clinically meaningful increase in prostate cancer risk, mainly due to substantial between-study heterogeneity, an effect of heavier alcohol drinking on high-grade disease in particular cannot be excluded. Between-study heterogeneity is likely to be generated by methodological limitations at individual study level, which could either mask true underlying effects, or generate spurious results. These limitations include the sick-quitter effect (a form of reverse causation), associative selection bias, residual confounding and measurement error (typical of all observational studies of alcohol effects), as well as detection bias (specific to prostate cancer, since prostate-specific antigen (PSA) screening is associated with health-related behaviors,^12^,^13^ and PSA itself might be affected by alcohol independently of prostate cancer). Moreover, it is possible that many studies were underpowered to detect small effects.

The aim of this study was to investigate the association of alcohol consumption from self-reported data with PSA levels and risk of PSA-detected prostate cancer, both overall and subgrouped according to stage and grade. We analyzed results from a large population-based case-control study nested within the PSA-testing phase of a randomized controlled trial for the treatment of localized prostate cancer—the Prostate testing for cancer and Treatment (ProtecT) trial. This study design overcomes some of the problems complicating the interpretation of the current epidemiological evidence: (*i*) it minimizes reverse causation in the forms of both the sick-quitter effect and recall bias or other differential information biases (because exposure information is collected before PSA test results become available); (*ii*) the fact that all cases are PSA-detected protects against gross levels of detection bias (caused for example by more health-conscious men undergoing (more frequent) PSA testing, whereas here all participants underwent PSA testing); and (*iii*) the large sample size allows the investigation of modest effects.

## Material and Methods

### Study design and outcomes definition

Participants in this study were recruited in the PSA-testing phase of the ProtecT study. Details of the protocol have been described elsewhere.^14^ Briefly, men aged 50–69 years in general practices located around nine UK cities were invited to attend a nurse-led prostate check clinic and, if they consented, to have a PSA test. Participants with a single raised PSA test result (≥3.0 ng/ml) were invited to attend the center's urology department for digital rectal examination, repeat PSA test, and 10-core trans-rectal ultrasound-guided biopsy.

**Figure 1:**
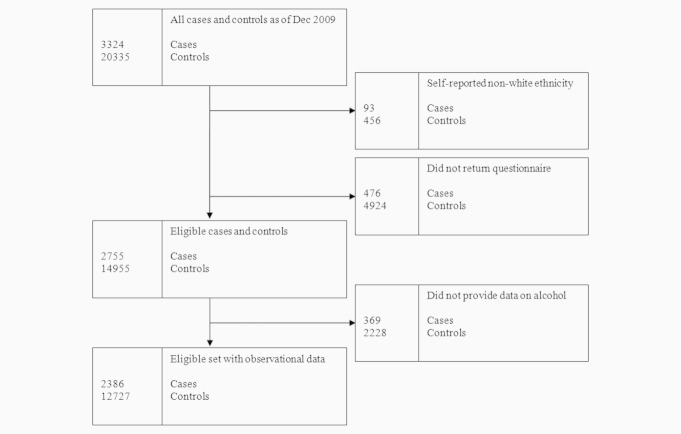
Flow-chart of exclusions and final data-sets of study participants–ProtecT nested case-control.

Eligible cases were the 3,324 men aged 50–69 with histologically-confirmed prostate cancer, detected among the 111,348 men undergoing PSA-testing between 11.2001 and 12.2009 [of which 10,297 (11%) had raised PSA]. Prostate cancer cases were classified into localized or advanced stage according to TNM clinical staging,^15^ and into low- and high-grade according to Gleason scores following review of histological material,^16^ as previously described.^17^ All participants in the ProtecT cohort who had no evidence of prostate cancer were eligible for selection as controls. These included all men with a PSA<3.0 ng/ml or PSA ≥3.0 ng/ml and a negative biopsy. Cases were frequency-matched (incidence-density sampling) to ≤6 controls by age (5-year bands) and general practice. As the clinics were held and completed in each general practice in turn, matching for general practice automatically matched for the calendar date of recruitment. Men of ethnicity other than white were a small minority and were excluded. Overall, 2,386 (69% of the total) men with histologically confirmed prostate cancer and 12,727 controls (63% of the total) returned the questionnaire and completed the section on alcohol during the prostate check clinic (Fig. 1).

Study participants gave fully informed consent in writing for the use of their data for research purposes. The Trent Multicentre Research Ethics Committee approved the study.

### Data on alcohol and related phenotypes (potential confounding factors)

Participants were asked to complete a health and lifestyle questionnaire after the check clinic but before their PSA result was available. This included a section on alcohol, with questions on usual frequency and amount consumed over the previous year, changes in consumption over the previous 10 years, and amount drunk in the previous week, and distinguished between types of alcoholic drinks. Usual weekly alcohol intake was obtained by combining intake amount and frequency separately for beer and spirits/wine (weekly number of half pints of beer and weekly number of glasses of wine and units of spirits). This was then converted into standard UK units (1 unit = 8 g of ethanol, corresponding to ∼ 10 ml of alcohol) by assuming the following volumes and alcohol contents: half pint of beer–284 ml, 3.5%; glass of wine–125 ml, 12%; standard pub measure of spirit–35 ml, 40% (http://www.cks.nhs.uk/alcohol_problem_drinking/background_information/definition/unit_of_alcohol). People self-reporting only occasional alcohol consumption (special occasions) were excluded from the computation of usual weekly alcohol intake. The continuous variable “weekly units of alcohol consumed” only took positive values and was used in analyses investigating the dose-response effect of alcohol on PSA levels and on PSA-detected prostate cancer among current drinkers. For the purpose of analysis, another variable with five categories was derived that distinguished men who did not drink, men drinking on special occasions only, and men who were current drinkers, divided into thirds of weekly alcohol consumption (based on the distribution of weekly drinking among controls). Additional alcohol behavior variables were drinking most days of the week over the past year (*versus* more occasional/sporadic drinking), referred to as “regular drinking,” and binge drinking, defined as usually consuming at least 5 drinks per drinking session. The traditional threshold of 5 or more drinks (= 5+ units) per drinking occasions for the classification of binge drinking was chosen based on recent evidence confirming its validity in predicting alcohol-related risk.^18^

Self-reported data on current diet, lifestyle, history of comorbidities and occupation were also obtained from the questionnaires. Information on skin color, as proxy for ethnicity, was extracted during the nurse-led clinic, and >95% of the ProtecT cohort were recorded as “White”. During this visit, the nurse measured participants' standing and sitting height, their weight, waist and hips circumference, and diastolic and systolic blood pressure, following standard operating procedures.

### Statistical analysis

To test the association between PSA levels and dimensions of alcohol behavior capturing patterns and levels of consumption (*e.g*., binge drinking, regular drinking, and amount consumed), we fitted linear regressions to data from randomly selected controls from the ProtecT study. The outcome was first log-transformed; therefore, effect estimates of the association are expressed as ratios of geometric means (RGMs). For example, an RGM of 0.96 corresponds to a (geometric) mean PSA difference of −4%. To test the association between alcohol and risk of PSA-detected prostate cancer, we fitted conditional logistic regressions to the case-control data set, and effect estimates are expressed as odds ratios (OR). Finally, to test the association between alcohol and prostate cancer risk by stage and grade sub-types, we used multinomial logistic regressions and ran two separate sets of analyses, each with the outcome variable grouped into 3 categories: (*i*) controls, localized cases (stage T1 or T2; NXM0), and advanced cases (stage T3 or T4); (*ii*) controls, low-grade Gleason cases (Gleason sum < 7), and high-grade Gleason cases (Gleason sum ≥ 7). Heterogeneity in associations of alcohol with localized as compared with advanced stage or low-grade compared with high-grade cancers were tested using Wald tests. Effect estimates of the association are expressed as relative risk ratios (RRRs).

We investigated the possible confounding of current BMI and blood pressure, as well as history of hypertension, hypercholesterolemia and diabetes, on the alcohol-PSA and alcohol-prostate cancer associations by performing likelihood ratio tests between nested models with and without them as covariates.

All analyses were matched on (prostate cancer risk) or adjusted for (PSA levels, prostate cancer sub-types) the design variables used for matching controls to cases (age in 5-year groups, center and date of recruitment and clinic visit), and additionally for age in years as a continuous variable, since both alcohol behavior and the outcomes are strongly age-dependant. All statistical tests were two-sided.

### Sensitivity analyses

Sensitivity analyses were performed to assess robustness of the alcohol-PSA and alcohol-prostate cancer associations based on self-reported data. Possible differential effects of recent *versus* usual drinking and of different beverage types on outcomes were investigated by comparing (*i*) estimates based on questions referring to the last 7 days only *versus* usual consumption over the past year, and (*ii*) estimates referring to total consumption, alcohol from beer only, and alcohol from wine and spirits combined (over past year). We also attempted to detect the presence of and adjust for potential bias caused by former heavy drinkers reducing their alcohol intake in middle-age because of comorbidities that may be alcohol-related (sick-quitter effect). To explore the possible impact of such bias, we performed sensitivity analyses by stratifying the analyses of the association between alcohol and PSA and prostate cancer risk according to whether men reported changing their drinking behavior in the last 10 years.

## Results

A description of selected characteristics is presented in Table 1. Cases were much more likely to have a positive family history of prostate cancer, more likely to have a positive history of hypertension, and less likely to have been diagnosed with diabetes, engage in strenuous exercise, and drink most days of the week.

**Table 1:** Characteristics of cases and controls included in the analysis of alcohol and prostate cancer risk

	Controls		Cases	
	*N*^1^	Mean (SD) or %	*N*^1^	Mean (SD) or %
Age (years)	12,727	62.0 (5.0)	2,386	62.0 (5.0)
Height (cm)	12,373	176.0 (6.6)	2,337	176.2 (6.7)
BMI (kg/m^2^)	12,134	27.0 (3.7)	2,287	26.9 (3.5)
Waist (cm)	11,034	96.4 (9.9)	2,085	96.4 (9.5)
Systolic blood pressure (mm Hg)	10,737	149.4 (19.8)	2,032	151.3 (20.0)
Diastolic blood pressure (mm Hg)	10,737	85.2 (11.2)	2,032	86.3 (10.8)
History of hypertension (yes *versus* no)	11,289	85.3%	2,142	86.7%
History of diabetes (yes *versus* no)	10,528	8.2%	1,990	6.3%
History of hypercholesterolemia (yes *versus* no)	10,756	30.4%	2,034	30.6%
Manual occupation (*versus* nonmanual)^2^	11,161	52.6%	2,171	53.2%
Strenuous exercise (yes *versus* no)	9,135	41.6%	1,730	39.1%
Vitamin supplement use (yes *versus* no)	12,357	50.2%	2,316	51.7%
Family history of prostate cancer (yes *versus* no)	12,727	4.8%	2,386	7.4%
Lifelong teetotalers (yes *versus* no)	12,725	2.6%	2,386	3.1%
Binge drinking^3^ (yes versus no)	12,613	12.0%	2,374	11.5%
Drinking most days (yes *versus* no)	12,727	39.0%	2,386	37.6%
Weekly alcohol consumption (units)^4^	11,053	13.6 (1.6)	2,079	13.4 (1.7)

1 Number with complete data.

2 Nonmanual occupation includes codes for: professional, managerial, nonmanual, and skilled nonmanual. Manual occupation includes codes for: manual and skilled manual, semiskilled, and unskilled manual.

3 Regularly consuming 5+ units/occasion.

4 Geometric mean. Abbreviations SD, standard deviation; BMI, body mass index.

### Patterns of consumption—Binge drinking and regular drinking

Men drinking most days had lower PSA values than men drinking less frequently (RGM 0.96, 95% CI: 0.93–0.98), and the same was true for those binge drinking regularly (Table 2). Results for risk of total prostate cancer were in the same direction, with lower risk for those binge drinking and drinking regularly, although confidence intervals (CI) were wider and also compatible with the opposite effect (Table 2). On the basis of results of multinomial logistic regressions and tests for the difference in effect estimates for different cancer sub-types, there was no clear evidence of an association of binge drinking or regular drinking with prostate cancer clinical stage at diagnosis (Table 2). Men drinking on most days seemed less likely to be diagnosed with advanced disease as compared to those who did not (RRR 0.77, 95% CI: 0.58–1.04), but this was based on just over 200 cases. There was a suggestion that binge drinking was associated with lower risks of low-grade disease, but no substantial change or perhaps a small increase in risk of high-grade disease, and a similar trend was noted for drinking most days (Table 2).

**Table 2:** Associations of dimensions of alcohol drinking behavior with PSA and prostate cancer risk

	Ratio o means or rela ratiosf geometric, odds ratios tive risk		*p*^1^
PSA levels – controls only^2^ (12,727 controls)			
Binge drinking^3^	0.96	(0.92; 1.00)	–
Drinking most days	0.96	(0.93; 0.98)	–
Total prostate cancer^4^ (2,386 cases)			
Binge drinking^3^	0.93	(0.81; 1.08)	–
Drinking most days	0.95	(0.87; 1.04)	–
Localized stage prostate cancer^5^ (1,914 cases)			
Binge drinking^3^	0.94	(0.81; 1.10)	–
Drinking most days	0.97	(0.88; 1.07)	–
Advanced stage prostate cancer^5^ (206 cases)			
Binge drinking^3^	0.93	(0.60; 1.45)	0.955
Drinking most days	0.77	(0.58; 1.04)	0.156
Low-grade prostate cancer^5^ (1,603 cases)			
Binge drinking^3^	0.87	(0.73; 1.03)	–
Drinking most days	0.91	(0.81; 1.01)	–
High-grade prostate cancer^5^ (775 cases)			
Binge drinking^3^	1.09	(0.87; 1.37)	0.097
Drinking most days	1.02	(0.88; 1.19)	0.191

Analysis of 2,386 cases and 12, 727 controls.

1 Test for difference in the effect estimates for localized versus advanced and low-grade versus high-grade prostate cancer.

2 Ratios of geometric means and 95% confidence intervals from linear regression, adjusted for the design variables on which controls and cases were matched–additionally adjusted for age as continuous variable.

3 Regularly consuming 5+ units/occasion.

4 Odds ratios and 95% confidence intervals from conditional logistic regression–strata defined by age, center and date of recruitment, and clinic visit–additionally adjusted for age as continuous variable.

5 Relative risk ratios and 95% confidence intervals from logistic regression, adjusted for the design variables on which controls and cases were matched—additionally adjusted for age as continuous variable. For definitions of localized/advanced stage and low/high-grade, see methods section. Abbreviations PSA, prostate-specific antigen.

### Dose-response: Alcohol intake in drinkers

There was evidence of a small dose-response type reduction in PSA levels with increasing alcohol consumption (RGM 0.98, 95% CI: 0.98–0.99 per 10 units/week increase) (Table 3). Results for total prostate cancer risk were similar in direction but less precisely estimated (Table 3). Also, the risk of both localized and advanced cancer seemed to reduce with increasing alcohol consumption when comparing men in the top third of the distribution to men in the bottom third (Table 3). There was strong evidence that the dose-response effects for weekly alcohol consumption and prostate cancer risk differed by grade at diagnosis (*p* = 0.004), with a suggestion that high-grade cancer risk was higher in heavier drinkers (RRR 1.04, 95% CI: 0.99–1.08), but the opposite was true for low-grade disease (RRR 0.96, 95% CI: 0.93–0.99).

**Table 3:** Association of weekly alcohol consumption with PSA and prostate cancer risk

	1^st^ third (0.1–9.8 units/week)	2^nd^ third (9.9–19.7 units/week)	3^rd^ third(19.8–112 units/week)	Dose-response^1^	*p*_*trend*_	*P*^2^
PSA levels–controls only^3^ (11,086 controls)	1	0.97 (0.94; 1.01)	0.92 (0.89; 0.95)	0.98 (0.98, 0.99)	<0.001	–
Total prostate cancer^4^ (2,083 cases)	1	0.94 (0.84; 1.06)	0.92 (0.82; 1.04)	0.99 (0.96; 1.01)	0.347	–
Localized stage prostate cancer^5^ (1,676 cases)	1	0.97 (0.85; 1.09)	0.93 (0.81; 1.05)	0.99 (0.96; 1.02)	0.573	–
Advanced stage prostate cancer^5^ (178 cases)	1	0.87 (0.61; 1.25)	0.86 (0.59; 1.23)	0.95 (0.87; 1.04)	0.238	0.338
Low-grade prostate cancer^5^ (1,400 cases)	1	0.97 (0.85; 1.11)	0.84 (0.73; 0.96)	0.96 (0.93; 0.99)	0.017	–
High-grade prostate cancer^5^ (675 cases)	1	0.86 (0.71; 1.05)	1.11 (0.92; 1.34)	1.04 (0.99; 1.08)	0.094	0.004

Analysis of 2,083 cases and 11,086 controls who were drinking alcohol at the time of assessment.

1 Per 10 units/week increase.

2 Test for difference in the effect-estimates for localized *versus* advanced and low-grade *versus* high-grade prostate cancer.

3 Ratios of geometric means and 95% confidence intervals from linear regression, adjusted for the design variables on which controls and cases were matched–additionally adjusted for age as continuous variable.

4 Odds ratios and 95% confidence intervals from conditional logistic regression models, additionally adjusted for age as a continuous variable.

5 Relative risk ratios and 95% confidence intervals from logistic regression, adjusted for the design variables on which controls and cases were matched—additionally adjusted for age as continuous variable. For definitions of localized/advanced stage and low/high-grade, see methods section. Abbreviations PSA, prostate-specific antigen.

### Adjustment for potential confounding factors

In general, adjustment for current BMI and systolic blood pressure did not substantially change the effect estimates (model 2 when compared to model 1); however, adjustment for pre-existing conditions such as history of hypertension, hypercholesterolemia and diabetes (model 3 when compared to model 2) did attenuate the association of binge drinking with PSA levels (Table 4), and prostate cancer risk (Table 5). Moreover, fully-adjusted models showed evidence of lower prostate cancer risk but not of lower PSA in nondrinkers as compared to drinkers in the first thirds of the distribution (OR 0.79, 95% CI: 0.64–0.99), but this effect was less pronounced in models 1 and 2 (Table 5).

**Table 4:** Association of alcohol drinking with PSA levels in controls

		Model 1^1^		Model 2^2^		Model 3^3^	
Exposure	*N*_co_	RGM	(95% CI)	RGM	(95% CI)	RGM	(95% CI)
Binge drinking^4^ (Yes *versus* No)	1,518	0.96	(0.92; 1.00)	0.98	(0.94; 1.03)	1.00	(0.95; 1.05)
Drinking most days (Yes *versus* No)	4,951	0.96	(0.93; 0.98)	0.95	(0.93; 0.98)	0.95	(0.92; 0.98)
Categories of consumption							
Nondrinker	1,028	0.94	(0.89; 0.99)	0.97	(0.92; 1.03)	0.97	(0.92; 1.03)
Special occasions only	613	1.01	(0.95; 1.08)	1.02	(0.95; 1.10)	1.05	(0.98; 1.13)
1st third of weekly intake (0.1–9.8 units/week)	3,805	1	–	1	–	1	–
2nd third of weekly intake (9.9–19.7 units/week)	3,739	0.97	(0.94; 1.01)	0.98	(0.95; 1.02)	0.98	(0.94; 1.02)
3rd third of weekly intake (19.8–112 units/week)	3,542	0.92	(0.89; 0.95)	0.92	(0.88; 0.95)	0.92	(0.88; 0.96)

1 Adjusted for age, center and date of recruitment and clinic visit.

2 Adjusted for variables in Model 1, and current BMI and measured systolic blood pressure.

3 Adjusted for variables in Model 2, plus history of diabetes, history of hypertension and history of hypercholesterolemia.

4 Regularly consuming 5+ units/occasion. Abbreviations PSA, prostate-specific antigen; RGM, ratio of geometric means; CI, confidence interval; N_co_, number of controls.

**Table 5:** Association of alcohol drinking with prostate cancer risk comparing cases and controls

		Model 1^1^		Model 2^2^		Model 3^3^	
Exposure	*N*_*ca*_/*N*_*co*_	OR	(95% CI)	OR	(95% CI)	OR	(95% CI)
Binge drinking^4^ (Yes *versus* No)	272/1,518	0.93	(0.81; 1.08)	0.96	(0.82; 1.13)	0.99	(0.84; 1.17)
Drinking most days (Yes *versus* No)	897/4,951	0.95	(0.87; 1.04)	0.95	(0.86; 1.05)	0.94	(0.84; 1.05)
Categories of consumption							
Nondrinker	185/1,028	0.90	(0.75; 1.08)	0.91	(0.74; 1.11)	0.79	(0.64; 0.99)
Special occasions only	123/613	1.02	(0.83; 1.26)	1.09	(0.86; 1.37)	1.05	(0.82; 1.35)
1st third of weekly intake (0.1–9.8 units/week)	745/3,805	1	–	1	–	1	–
2nd third of weekly intake (9.9–19.7 units/week)	689/3,739	0.94	(0.84; 1.06)	0.94	(0.82; 1.06)	0.91	(0.80; 1.04)
3rd third of weekly intake (19.8–112 units/week)	644/3,542	0.92	(0.82; 1.04)	0.94	(0.82; 1.07)	0.91	(0.79; 1.04)

1 Conditional logistic regression—strata defined by age, center and date of recruitment and clinic visit—additionally adjusted for age as continuous variable

2 Additionally adjusted for current BMI and measured systolic blood pressure.

3 Additionally adjusted for variables in Model 2, plus history of diabetes, history of hypertension and history of hypercholesterolemia.

4 Regularly consuming 5+ units/occasion. Abbreviations OR, odds ratio; CI, confidence interval; N_ca_/N_co_, number of cases/number of controls.

### Sensitivity analyses

Estimates of the association of PSA levels and prostate cancer risk with weekly alcohol consumption, derived through either 7-day recall questions or average quantity and frequency questions referring to the previous 12 months, were in good agreement with each other, with 95% CIs largely overlapping (Figs. S1 and S2, Supporting Information online).

Dose-response effect estimates for total consumption, alcohol from beer only, and alcohol from wine and spirits combined were all similar and suggested that alcohol could lower PSA levels by 2–3% per 10 units/week increase in consumption (Table S1a, Supporting Information online). For prostate cancer risk, point estimates were in the same direction, but there was limited statistical evidence to show decreased risks specific to any beverage type (Table S1b, Supporting Information online).

Compared to analyses including the entire sample, restricting analyses to men who did not change drinking habits left most results unchanged. As expected, there was a suggestion of attenuated effects in men who changed drinking behavior in the last 10 years, as compared to men who did not, for both PSA levels and prostate cancer risk (Tables 6 and 7).

**Table 6:** Association of alcohol drinking with (a) PSA levels in controls, stratified according to changes in drinking behavior in the last 10 years

	Same drinking behavior			Changed drinking behavior		
Exposure	*N*_*co*_	RGM^1^	(95% CI)	*N*	RGM^1^	(95% CI)
Binge drinking^2^ (Yes *versus* No)	806	0.96	(0.91; 1.01)	650	0.97	(0.91; 1.03)
Drinking most days (Yes *versus* No)	2,738	0.96	(0.93; 1.00)	1,974	0.96	(0.92; 1.01)
Categories of consumption						
Nondrinker	704	0.93	(0.87; 0.99)	294	0.95	(0.86; 1.05)
Special occasions only	431	1.00	(0.93; 1.08)	163	0.99	(0.87; 1.12)
1st third of weekly intake	2,649	1	–	1,019	1	–
2nd third of weekly intake	2,224	0.96	(0.92; 1.01)	1,376	1.01	(0.95; 1.07)
3rd third of weekly intake	1,954	0.91	(0.87; 0.95)	1,417	0.97	(0.90; 1.04)

1 Adjusted for age, center and date of recruitment and clinic visit.

2 Regularly consuming 5+ units/occasion.

Abbreviations PSA, prostate-specific antigen; RGM, ratio of geometric means; CI, confidence interval; *N*_co,_ number of controls.

**Table 7:** Association of alcohol drinking with prostate cancer risk comparing cases and controls, stratified according to changes in drinking behavior in the last 10 years

	Same drinking behavior			Changed drinking behavior		
Exposure	*N*_*ca*_/*N*_*co*_	OR^1^	(95% CI)	*N*_*ca*_/*N*_*co*_	OR^1^	(95% CI)
Binge drinking^2^ (Yes *versus* No)	147/806	0.94	(0.77; 1.15)	114/650	0.89	(0.70; 1.13)
Drinking most days (Yes *versus* No)	500/2,738	0.93	(0.82; 1.05)	348/1,974	0.98	(0.82; 1.16)
Categories of consumption						
Nondrinker	132/704	0.92	(0.74; 1.14)	44/294	0.81	(0.55; 1.19)
Special occasions only	95/431	1.10	(0.86; 1.41)	25/163	0.86	(0.53; 1.40)
1st third of weekly intake	536/2,649	1	–	182/1,019	1	–
2nd third of weekly intake	406/2,224	0.92	(0.79; 1.06)	259/1,376	1.00	(0.80; 1.26)
3rd third of weekly intake	363/1,954	0.92	(0.79; 1.07)	247/1,417	0.93	(0.74; 1.17)

1 Conditional logistic regression—strata defined by age, center and date of recruitment and clinic visit—additionally adjusted for age as continuous variable

2 Regularly consuming 5þ units/occasion.

Abbreviations OR, odds ratio; CI, confidence interval; *N*_ca_/*N*_co_, number of cases/number of controls.

## Discussion

### Summary of results

On the basis of this large case-control study nested in the ProtecT trial, there was some evidence that increasing alcohol consumption and frequency of drinking were associated with lower PSA and weaker evidence that they were also associated with lower risk of PSA-detected prostate cancer among current drinkers, with generally wide confidence intervals. This was attributable to a decrease in low Gleason grade tumors, the most common cancer sub-type detected through PSA testing, whereas a small increase was observed for the more aggressive high-grade tumors, in line with the suggestion of a small increase in risk of high-grade cancer and a decrease for low-grade cancer for binge drinkers. There was limited evidence of a differential association with alcohol according to tumor TNM stage.

There was a tenuous suggestion of a nonlinear association of alcohol with the outcomes, as nondrinkers tended to have lower PSA and lower risk of PSA-detected prostate cancer as compared to light drinkers. However, confidence intervals were somewhat overlapping and occasionally compatible with no effect. Estimates were almost unchanged when restricted to men reporting not to have changed drinking habits in the past 10 years (*i.e.*, no evidence of sick-quitter effect).

### Comparison with previous literature and interpretation of results

The small reduction in risk of PSA-detected prostate cancer observed in this study (OR 0.985, 95% CI: 0.96–1.01 per 10 units/week increase) contrasts with most of the published evidence to date. Meta-analytical results based on two large systematic reviews of the literature up to 2006 and 2007 are compatible with no effect, but point towards an increase in prostate cancer risk for increasing weekly consumption (RR 1.04, 95% CI: 1.00–1.10,^5^ and RR 1.03, 95% CI: 0.79–1.34^4^).

The current study is the only one in the literature on alcohol and prostate cancer risk whose prostate cancer cases were identified based on a PSA threshold of 3 ng/ml. Discrepancies between our result and previous prospective studies could be due to lower chances of a cancer diagnosis for the heaviest drinkers, because of their lower PSA levels. This is one of the first population-based studies, and the largest to date, to investigate the alcohol-PSA association. Previously two cross-sectional studies did not find strong evidence in support of an association.^7^,^19^ The observed negative association is in line with published evidence of an inverse association of alcohol with benign prostate hyperplasia (BPH),^20^ possibly underpinned by mechanisms independent of prostate cancer. It is not clear whether the relationship is causal, or due to the presence of confounding, or to reverse causation. The latter might be an issue since most observations of the alcohol-PSA and alcohol-BPH association are cross-sectional, and it is possible that men with increased PSA because of BPH and urinary symptoms might reduce their alcohol consumption. However, the prevalence of lower urinary tract symptoms in this unselected population-based sample was low,^21^ and therefore unlikely to have substantially impacted on men's drinking behavior.

Results from this study are still compatible with the hypothesis that alcohol could increase prostate cancer risk itself, as suggested by the observations of an increase in high-grade disease in heavy drinkers. The stronger evidence of association with alcohol drinking observed for high-grade tumors could be due to the fact that they are less likely to be affected by detection bias, or to an underlying different etiology of less aggressive and more aggressive disease. The ProtecT data showed a suggestion of a threshold effect in the association of alcohol with high-grade prostate cancer, with a RRR of 1.11 (95% CI: 0.92–1.34) comparing the top third to the bottom third of alcohol consumption. This is in line with what had been suggested by recent prospective studies examining the risk of high-grade disease.^7^–^9^ The highest drinking category in this study comprised of men drinking 20+ alcohol units/week, equivalent to ∼ 24+ g of ethanol/day. This group therefore includes more moderate drinkers as compared to the top category used in the European Prospective Investigation into Cancer and Nutrition study (60+ g/day, RR 1.17, 95% CI: 0.81–1.67)^8^ and in the Prostate Cancer Prevention Trial (50+ g/day, RR 2.01, 95% CI: 1.33–3.05)^7^, but is comparable to the top category of the Health Professionals Follow-up Study (20+ g/day, RR 1.72, 95%CI: 0.88–3.36).^9^

### Potential mechanisms

The existence of a threshold effect with increased risk observed in heavy drinkers but not in light or moderate drinkers is biologically plausible. High acetaldehyde concentrations could promote carcinogenesis in prostatic epithelial tissue.^22^ Acetaldehyde is mostly produced in the liver by alcohol dehydrogenases, and can reach the prostate through the bloodstream. Once in the prostate, it would be difficult to clear acetaldehyde because acetaldehyde dehydrogenase enzymatic activity is low in prostatic epithelial tissue (http://bioinfo.wilmer.jhu.edu/tiger/db_gene/ALDH2-index.html). Only very heavy drinking could result in high enough intra-prostatic acetaldehyde concentrations to be carcinogenic (*i.e.*, 40–1000 μ*M*, based on *in vitro* experiments,^23^ with *in vitro* acetaldehyde levels correlating well with *in vivo* levels^24^). Whereas systemic acetaldehyde concentrations following alcohol ingestion are usually in the range of 1–5 μ*M*,^25^ in alcoholics with blood alcohol concentrations >80 m*M* they have been found to reach 40 μ*M*, even 10–12 h after the last drink.^26^ Also, it is currently believed that chronic exposure to oxidative stress, which can be the result of chronic heavy drinking in combination with antioxidant deficiency or malabsorption, may have a carcinogenic effect in the prostate gland and affect both tumor aggressiveness and rate of progression.^27^ Both these mechanisms arise from (chronic) heavy alcohol drinking, and therefore suggest a threshold effect rather than a dose-response relationship.

### Strengths and limitations

Strengths of this study design include adequate power provided by the large sample size, no recall bias, minimal sick-quitter effect and reduced detection bias. Reverse causation, in the form of the sick-quitter effect or otherwise, could explain why moderate and heavy drinkers have lower PSA than light drinkers. It is in fact possible that men with high PSA caused by an enlarged prostate and at higher risk of urinary tract symptoms would have cut down on alcohol, since alcohol is one of the bladder irritants, which could cause urinary retention.^20^ However, in analyses where the sample was restricted to men who had not modified their drinking habit in the past 10 years, the results were almost unchanged, suggesting that reverse causation is an unlikely explanation of our observations, and the prevalence of lower urinary tract symptoms was low.^21^ A further advantage is that complete data on PSA levels for all participants allowed estimation of the alcohol-PSA association.

The use of a PSA threshold (3 ng/ml) for initiating the diagnostic process could complicate the interpretation of results. On the one hand, the possibility that some of the controls might have undiagnosed prostate cancer might attenuate the estimated effect. On the other, since alcohol behavior and associated factors could affect PSA levels independently of prostate cancer, this could introduce a bias resulting in apparent changes in risk of PSA-detected prostate cancer, in particular for low-grade tumors, as discussed in the above paragraph. However, the impact of detection bias is likely to be limited for associations between alcohol and risk of high Gleason-grade prostate cancer. Where all individuals undergo PSA testing, like in ProtecT, high-grade tumors will be less affected by a possible effect of alcohol on PSA, since they are usually characterized by high levels of the marker.^28^ Also, in situations where not everyone is PSA tested, as is the case in many studies from the literature, detection bias is minimized for these tumor sub-types as they are more likely to progress to advanced and metastatic stage and become symptomatic quicker,^29^,^30^ and to be diagnosed regardless of PSA testing. Therefore, associations found between alcohol and high-grade tumors are generally more robust than those for total prostate cancer or low-grade disease.

Limitations of this study include confounding, selection bias, measurement error and to some degree recall bias. Confounding offers a particularly important challenge in the context of prostate cancer, as on the one hand alcohol drinking clusters with many other behaviors detrimental to health, and on the other little is known about environmental and lifestyle causes of prostate cancer,^5^ other than, for example, smoking does not seem to be one of these.^31^ In this scenario, speculation about residual confounding is common. We attempted adjustment for potentially confounding factors and generally found similar effect estimates; however, only quasi-experimental designs such as Mendelian randomization could reasonably exclude a major impact of confounding on the results.^32^ Selection bias might occur if heavy drinking study participants were self-selected and generally healthier (implying lower cancer risk) than nonparticipants, but generally such selection would not apply to moderate drinkers. However, this does not fit in with the observed dose-response association between alcohol and PSA, but would probably result in a scenario with a marked reduction of risk in heavy drinkers only as compared to light drinkers, and similar risk for moderate and light drinkers. If nonparticipation in the ProtecT study depended on previous PSA testing and/or prostatic symptoms, this could introduce another source of bias; however, PSA testing is not common place in the UK. Nondifferential measurement error was likely to be minimized by the choice of light drinkers over current nondrinkers as the reference category to improve internal validity of comparisons between groups of drinkers, and by the fact that participants displayed regular drinking behaviors, as evidenced by similar effects observed for recent and usual drinking across the entire distribution of alcohol intake. As for recall bias, there remains the possibility that men with prostate cancer symptoms reported their alcohol consumption differently from healthier men, or were more/less likely to fill in their questionnaires, although it is yet to be investigated whether in the ProtecT study response rates depended on PSA levels, a proxy for symptoms.

### Impact/implications

The nature of the association between alcohol consumption and prostate cancer is extremely important, as robust evidence on potentially modifiable risk factors for this disease is currently lacking.^33^ This is despite the fact that age, ethnicity and genetic/inherited factors can only predict 65% of disease,^34^ whereas international comparisons show higher incidence and mortality in wealthier countries and a positive correlation with elements of Western diets,^35^ and migrant studies suggest that low-risk populations quickly acquire higher prostate cancer incidence rates when moving to high-risk countries.^36^,^37^

If quasi-experimental studies can confirm the present results to be causal, prostate cancer risk will be added to the many long-term health risks of heavy drinking. Strategies to reduce the prevalence of drinking over the recommended amount will then also result in a small reduction in high-risk, poorer prognosis prostate cancer. For example, if the 30% of British men in this general population sample who currently drink more than 21 units/week were to cut down to less than 10 units/week, there could be a reduction in high-grade prostate cancer of around 3%, assuming a RR of 1.1 such as the one estimated in the present study. This is a modest effect, however, keeping alcohol consumption low throughout adulthood would be more effective in terms of prostate cancer prevention, given the long latency of prostate cancer and the suggestion that alcohol might act as a tumor initiator through procarcinogens activation, as well as a promoter.^38^

Establishing whether alcohol drinking causally influences PSA would help answering the question of how many more or fewer prostate cancer cases would be identified should drinking habits change in the population, at least as long as PSA testing remains widespread. On the other hand, the observation that men drinking heavily have lower PSA than men drinking lightly might have public health implications, even if not causal, provided that it is confirmed by independent studies. In the clinical setting, heavy alcohol consumption could be used as a marker identifying a group of men in which some cancers might be missed. The corresponding increase in mortality could be evaluated in current PSA screening trials, and these results might lead to recommending different PSA thresholds depending on usual and recent alcohol consumption.

## Conclusion

These results support the hypothesis that heavy alcohol drinking causes a small increase in risk of high-grade prostate cancer, and are generalizable to European-origin populations with widespread use of PSA testing. However, no firm conclusion can be reached on the nature of the effect of alcohol on low-grade cancers and on PSA in the absence of independent replication, preferably among large cohorts of men with a variety of grades and stages of prostate cancer.

## Abbreviations

BMI: body mass index
BPH: benign prostate hyperplasia
CI: confidence interval
OR: odds ratio
ProtecT: Prostate testing for cancer and Treatment
PSA: prostate-specific antigen
RGM: ratio of geometric means
TNM: tumor-node-metastasis
