# Supplementary material for: Alcohol consumption and PSA-detected prostate cancer risk—A case-control nested in the ProtecT study
Source: Int J Cancer. 2012 Oct 25;132(9):2176–85. doi: 10.1002/ijc.27877 (PMC3786564; doi:10.1002/ijc.27877)
Supplement: Supplementary file 1 [file ijc0132-2176-SD1.doc]

## Acknowledgements

This work was supported by the National Cancer Research Institute (administered by the Medical Research Council - MRC), which provided funding through the ProMPT (Prostate Mechanisms of Progression and Treatment) collaborative, and the UK National Institute of Health Research Health Technology Assessment (NIHR HTA) Programme (grant numbers 96/20/06, 96/20/99 to JLD, FCH and DEN). The work reported in this article was undertaken during the tenure of a UK MRC Special Training Fellowship awarded to LZ (grant number G0501864/76656).

The views and opinions expressed therein are those of the authors and do not necessarily reflect those of the HTA programme, NIHR, National Health Service or the Department of Health. The sponsors had no role in the analysis, the interpretation of the results, the preparation of the manuscript, or the decision to submit the manuscript for publication. The authors declare that they have no conflict of interest.

The authors would like to acknowledge the tremendous contribution of all members of the ProtecT study research group, and especially the following who were involved in this research. Research nurses [Recruitment, sample collection and follow-up]: lead: Sue Bonnington, Lynne Bradshaw, Debbie Cooper, Emma Elliott, Pippa Herbert, Peter Holding, Joanne Howson, Mandy Jones, Teresa Lennon, Norma Lyons, Hilary Moody, Claire Plumb, Tricia O’Sullivan, Liz Salter, Sarah Tidball, Pauline Thompson; others: Tonia Adam, Sarah Askew, Sharon Atkinson, Tim Baynes, Jan Blaikie, Viv Breen, Sean Bryne, Jo Bythem, Jenny Clarke, Jenny Cloete, Susan Dark, Gill Davis, Rachael De La Rue, Elspeth Dewhurst, Anna Dimes, Nicola Dixon, Penny Ebbs, Ingrid Emmerson, Jill Ferguson, Ali Gadd, Lisa Geoghegan, Alison Grant, Collette Grant, Catherine Gray, Rosemary Godfrey, Louise Goodwin, Susie Hall, Liz Hart, Andrew Harvey, Chloe Hoult, Sarah Hawkins, Sharon Holling, Alastair Innes, Sue Kilner, Fiona Marshall, Louise Mellen, Andrea Moore, Sally Napier, Julie Needham, Kevin Pearse, Anna Pisa, Mark Rees, Elliw Richards, Lindsay Robson, Janet Roxburgh, Nikki Samuel, Irene Sharkey, Michael Slater, Donna Smith, Pippa Taggart, Helen Taylor, Ayesha Thomas, Nicola Trewick, Claire Ward, Christy Walker, Ayesha Williams, Colin Woodhouse, Elizabeth Wyber and others. Local Investigators/clinicians: Prasad Bollina, Jim Catto, Andrew Doble, Alan Doherty, Garett Durkan, David Gillatt, Owen Hughes, Roger Kockelbergh, Howard Kynaston, Hing Leung, Edgar Paez , Alan Paul, Raj Persad, Philip Powell, Stephen Prescott, Derek Rosario, Hartwig Schwaibold, David Tulloch, Mike Wallace. Pathologists: Selina Bhattarai, Neeta Deshmukh, John Dormer, John Goepel, David Griffiths, Ken Grigor, Pat Harnden, Nick Mayer, Jon Oxley, Mary Robinson, Murali Varma, Anne Warren. Research, bio-repository and data management: Leila Ayandi, Lucy Brindle, Paul Brown, Simon Collin, Michael Davis, Dan Dedman, Elizabeth Down, Ewa Dudziec, Luke Ferguson, Anne George, Vriti Hansraj, Dawn Jordan, Selena Josephs, Rajeev Kumar, Adam Lambert, Athene Lane, Thomas Ludlam, Gemma Marsden, Luke Marsden, Steven Oliver, Josh Phillips, Jane Pritchard, Laura Proctor, Peter Shiarly, Martin Taylor, Emma Turner, Eleanor Walsh, Oliver Wilkinson, Valentina Wright. Administrative support: Susan Baker, Elizabeth Bellis-Sheldon, Chantal Bougard, Joanne Bowtell, Catherine Brewer, Nicholas Christoforou, Rebecca Clark, Susan Coull, Christine Croker, Rosemary Currer, Claire Daisey, Gill Delaney, Rose Donohue, Susan Fry, Jean Haddow, Susan Halpin, Belle Harris, Barbara Hattrick, Sharon Holmes, Helen Hunt, Vicky Jackson, Mandy Le Butt, Jo Leworthy, Tanya Liddiatt, Alex Martin, Jainee Mauree, Susan Moore, Gill Moulam, Jackie Mutch, Kathleen Parker, Christopher Pawsey, Michelle Purdie, Teresa Robson, Lynne Smith, Carole Stenton, Tom (Prasad Bollina, Sue Bonnington, Debbie Cooper, Andrew Doble, Alan Doherty, Emma Elliott, David Gillatt, Pippa Herbert, Peter Holding, Joanne Howson, Mandy Jones, Roger Kockelbergh, Howard Kynaston, Teresa Lennon, Norma Lyons, Hilary Moody, Philip Powell, Stephen Prescott, Liz Salter, Pauline Thompson). Department of Health disclaimer: The views and opinions expressed therein are those of the authors and do not necessarily reflect those of the Department of Health.

We especially thank Dr. Athene Lane, the ProtecT study coordinator, and Mr. Michael Davis for taking care of the ProtecT and ProMPT database and for sampling the control groups.
