# Supplementary material for: Alcohol consumption and PSA-detected prostate cancer risk—A case-control nested in the ProtecT study
Source: Int J Cancer. 2012 Oct 25;132(9):2176–85. doi: 10.1002/ijc.27877 (PMC3786564; doi:10.1002/ijc.27877)

Supplementary Material Online

Table S 1 Association between weekly alcohol consumption (in units) and a) PSA in control, b) prostate cancer risk comparing cases and controls. Geometric means of PSA and odds of prostate cancer and 95% confidence intervals by thirds of the distribution of alcohol consumption, test for trend and dose-response effect estimates.

a)

	Bottom third Mean† (95% CI)	Middle third Mean† (95% CI)	Top third Mean† (95% CI)	Dose-response*	P for trend
Total weekly alcohol units	1.1 (0.3, 4.1)	1.0 (0.3, 3.5)	0.97 (0.3, 3.4)	0.98 (0.98, 0.99)	<0.001
Units from beer	1.1 (0.3, 3.9)	1.0 (0.3, 3.5)	0.95 (0.3, 3.1)	0.97 (0.96, 0.99)	<0.001
Units from wine / spirits	1.1 (0.3, 4.1)	1.0 (0.3, 3.5)	0.99 (0.3, 3.6)	0.98 (0.97, 1.00)	0.017

† *Geometric mean*

* *Ratio of geometric means of PSA per 10 units increase a week, adjusted for age, centre and date of recruitment and clinic visit*

b)

	Bottom third Odds (95% CI)	Middle third Odds (95% CI)	Top third Odds (95% CI)	Dose-response*	P for trend
Total weekly alcohol units	0.20 (0.18; 1.21)	0.18 (0.17; 0.20)	0.18 (0.17; 0.20)	0.99 (0.96; 1.01)	0.347
Units from beer	0.20 (0.18; 0.21)	0.20 (0.18; 0.21)	0.18 (0.16; 0.19)	0.99 (0.95; 1.04)	0.771
Units from wine / spirits	0.20 (0.18; 0.22)	0.18 (0.16; 0.19)	0.18 (0.16; 0.20)	0.97 (0.93; 1.02)	0.196

* *Odds ratio of prostate cancer per 10 units increase a week, from conditional logistic regressions additionally adjusted for age as continuous variable*

Figure S 1 Association of prostate-specific antigen (PSA) with weekly alcohol drinking during the past year and the past week before PSA test. Ratios of geometric means (RGM) of PSA levels among controls and 95% confidence intervals (CI).

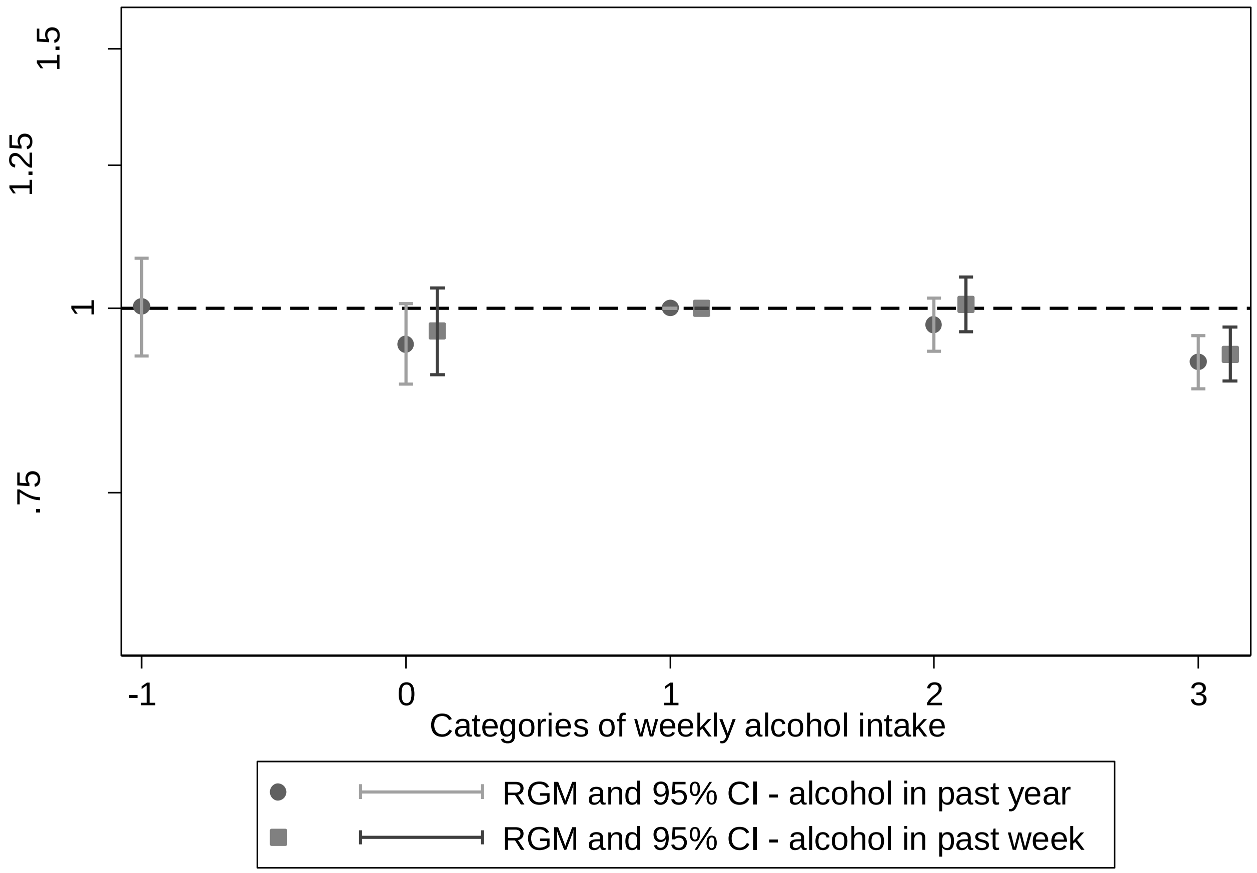

X-axis legend:

- 1: men drinking on special occasions only; 0: non-drinkers; 1 to 3 refer to thirds of weekly alcohol intake, with the bottom third (1) being the reference category.

Figure S 2 Association of prostate cancer risk with weekly alcohol drinking during the past year and the past week before PSA test. Odds ratios (OR) and 95% confidence intervals (CI) from conditional logistic regressions.

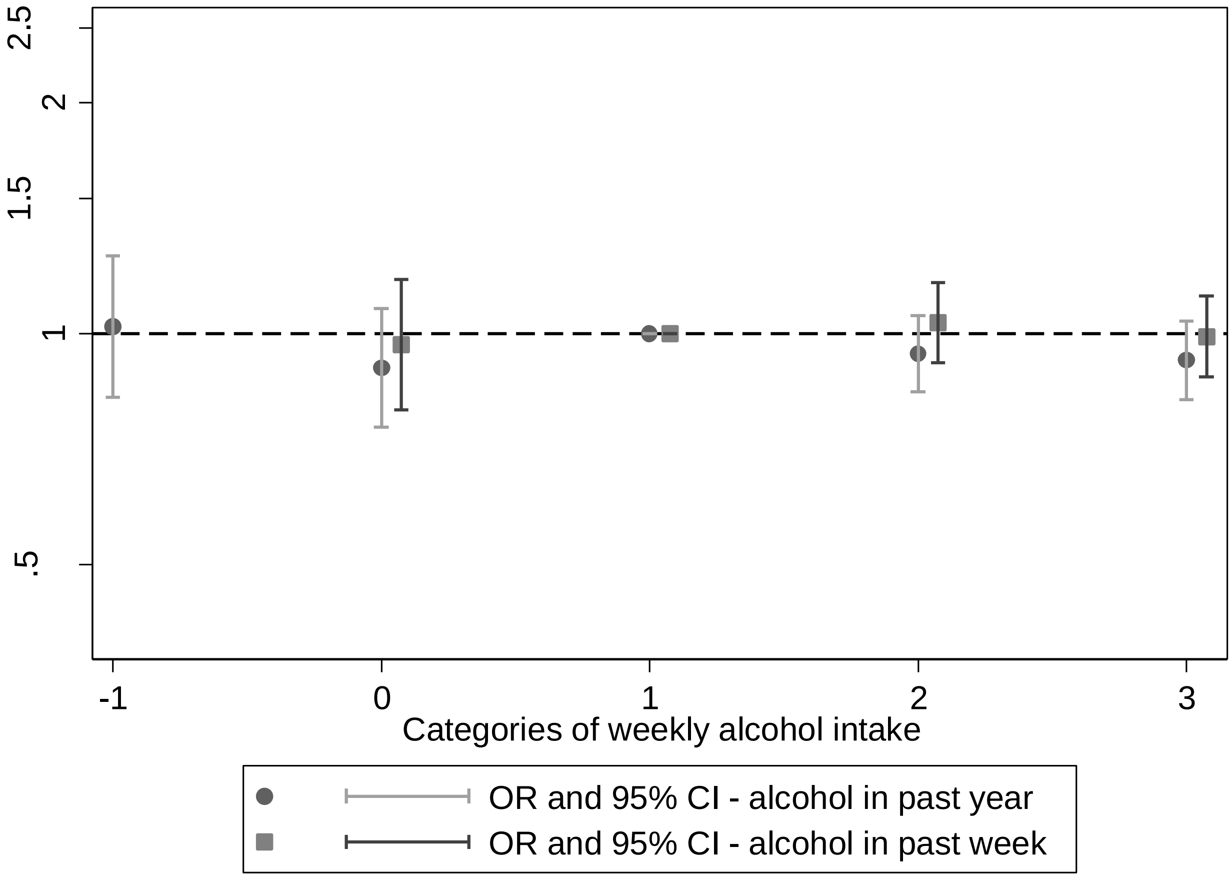

X-axis legend:

-1: men drinking on special occasions only; 0: non-drinkers; 1 to 3 refer to thirds of weekly alcohol intake, with the bottom third (1) being the reference category.

Figure S 3 Schematic representation of the hypothesised pathways behind the alcohol-PSA association. Scenarios without (top panel) and with (bottom panel) the "sick quitter" effect. Abbreviations: SES – socio-economic status; CVD – cardio-vascular disease; PSA – prostate-specific antigen.

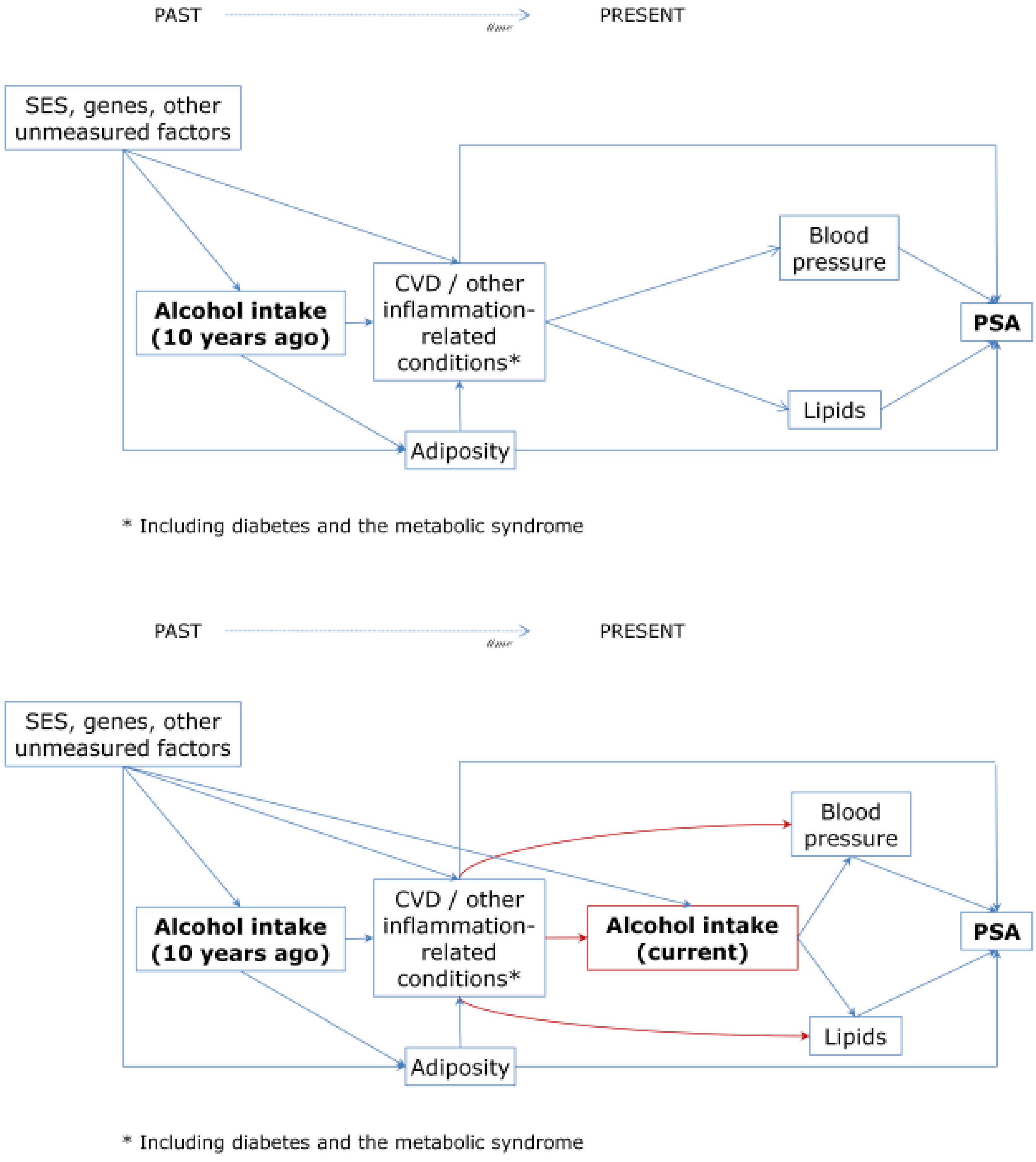

Supplement: Supplementary file 2 [file ijc0132-2176-SD2.pdf]
